# *Akkermansia muciniphila* Alleviates Sarcopenia in Senescence-Accelerated Mouse-Prone 8 Mice

**DOI:** 10.4014/jmb.2507.07001

**Published:** 2025-08-26

**Authors:** So-Hyun Park, Hee Soo Kim, Pyeong Geun Choi, Myeong Seon Jeong, Yang Hoon Huh, Jiyun Ahn, Chang Hwa Jung

**Affiliations:** 1 Aging and Metabolism Research Group, Korea Food Research Institute, Wanju 55365, Republic of Korea; 2 Department of Food Biotechnology, University of Science and Technology, Wanju 55365, Republic of Korea; 3 Electron Microscopy Research Center, Korea Basic Science Institute, Ochang 28119, Republic of Korea

**Keywords:** Aging, *Akkermansia muciniphila*, gut microbiome, sarcopenia, skeletal muscle

## Abstract

*Akkermansia muciniphila*, an intestinal bacterium, has garnered attention for its association with metabolic health and anti-inflammatory properties. However, its potential role in mitigating sarcopenia, particularly in the senescence-accelerated mouse-prone 8 (SAMP8) model, remains unexplored. In this study, we aimed to evaluate the potential effects of *A. muciniphila* supplementation on sarcopenia and its underlying mechanisms. Seven-month-old SAMP8 mice were administered *A. muciniphila* for 3 months. *A. muciniphila* supplementation enhanced grip strength and skeletal muscle mass, suppressed cellular senescence, improved the balance between protein degradation and synthesis, increased total ATP, and improved mitochondrial biogenesis. Analysis of the effects of *A. muciniphila* on gut microbiome using 16S rRNA sequencing showed that supplementation with *A. muciniphila* shifted the gut microbiota composition, alleviated gut dysbiosis, preserved gut barrier integrity, and reduced the protein expression of inflammatory cytokines in the intestine. Additionally, extracellular vesicles derived from *A. muciniphila* promoted myogenesis and suppressed dexamethasone-induced atrophy in C2C12 myoblasts. These findings suggest that supplementation with *A. muciniphila* mitigates sarcopenia by suppressing inflammation and improving the gut microenvironment, highlighting the potential of *A. muciniphila* as a new therapeutic candidate to treat sarcopenia.

## Introduction

With increased life expectancy and an aging population, the prevalence of age-related diseases, including sarcopenia, is rising globally [1]. Sarcopenia, a major geriatric disorder, is characterized by progressive loss of muscle mass and function with age [2]. Although the mechanisms underlying sarcopenia are not fully understood, factors such as cellular senescence, low-grade inflammation, inadequate nutrition, and imbalances in protein degradation and synthesis have been suggested to contribute to its pathophysiology [3]. Sarcopenia significantly diminishes the quality of life in older adults [4], with an increased risk of falls, fractures, and mortality [5, 6]. Furthermore, sarcopenia is associated with various age-related diseases such as obesity, Alzheimer’s disease, and cognitive impairment [7, 8]. Despite the significant effects of sarcopenia on healthy aging, no Food and Drug Administration-approved drugs for sarcopenia are currently available, highlighting the urgent need for new therapeutic approaches.

The gut microbiota has emerged as a promising therapeutic target for various age-related diseases, including sarcopenia [9]. Studies have shown a close relationship between sarcopenia and gut microbiota alterations with reduced microbial diversity and gut dysbiosis observed in affected individuals [10-12]. The alterations in the gut microbiota are thought to contribute to muscle atrophy by affecting the production of metabolites, such as short-chain fatty acids (SCFAs), and promoting inflammation. Disruption of the gut microbiota and loss of diversity can compromise the integrity of the intestinal barrier, leading to systemic inflammation characterized by elevated lipopolysaccharide, metabolic disorders, and decreased muscle function [13]. Therefore, targeting the gut microbiota through prebiotic, probiotic, and bacterial product interventions has garnered increased attention as a potential strategy for preventing sarcopenia. For instance, in a clinical study, prebiotic supplementation has been shown to increase muscle strength (measured with hand grip strength) in older adults [14]. Furthermore, probiotic supplementation reduced age-related decline in muscle mass and strength in senescence-accelerated mice by modulating gut microbiota, restoring the concentration of SCFAs, and attenuating inflammation levels [15]. Additionally, several other studies have reported the positive effects of probiotic supplementation on muscle mass and function in age-related diseases [16, 17], suggesting that probiotic intervention is a promising treatment for managing sarcopenia and disease-related muscle atrophy.

*Akkermansia muciniphila* is an intestinal bacterium first isolated from human feces in 2004 [18]. It comprises approximately 1–3% of the gut microbiota in healthy adults [19] and has emerged as “next-generation beneficial microorganism” due to its significant role in regulating intestinal and metabolic health [20]. In addition to its functional role as a live bacterium, *A. muciniphila* produces extracellular vesicles (EVs), which are nanoscale, spherical structures composed of lipid bilayer that can enter systemic circulation and modulate host physiological processes [21]. Recent studies have demonstrated that EVs derived from *A. muciniphila* (*Akk*-EVs) enhance gut barrier integrity and confer anti-inflammatory and metabolic benefits [22-24]. While several animal studies have reported that *A. muciniphila* supplementation supports muscle mass and function, the specific role of *Akk*-EVs in skeletal muscle maintenance and aging-related muscle decline remains largely unexplored [25, 26].

In this study, we aimed to investigate the effect and mechanism of action of *A. muciniphila* on sarcopenia in senescence-accelerated mouse-prone 8 (SAMP8) mice, a widely used model of accelerated aging. We evaluated skeletal muscle mass and function in SAMP8 mice after three months of *A. muciniphila* supplementation and explored its mechanism of action. We also investigated the effect of *Akk*-EVs on myogenesis.

## Methods

### *A. muciniphila* Culture

*A. muciniphila* was purchased from American Type Culture Collection (BAA-835; ATCC, USA) and cultured in a soy-peptone-based medium containing 20 g/l soy-peptone, 0.45 g/l K_2_HPO4, 0.45 g/l KH_2_PO4, 10 g/l yeast extract, 5.0 g/l N-acetyl-D-glucosamine, 5 g/l D-lactose, 2.5 g/l D-fructose, 4 g/l L-aspartic acid, 2 g/l sodium bicarbonate, 0.1 mg/l cyanocobalamin, and 0.5 g/l L-cysteine hydrochloride. The culture was carried out in an anaerobic chamber filled with 90% nitrogen, 5% carbon dioxide, and 5% hydrogen at 37°C. Bacteria were grown in media under anaerobic conditions on a gas phase of N_2_ at 37°C until the optical density at 600 nm reached 0.65 (stationary phase). Expected OD_600_ was determined by counting the viable cells in a bacterial sample using serial dilution and plate count methods. An OD_600_ value of 0.65 equals 5.0 × 10^9^ colony forming units (CFU)/ml.

### Isolation of Extracellular Vesicles Derived from *A. muciniphila*

*A. muciniphila* cultures were centrifuged at 13,000 ×*g* for 20 min at 4°C, and the supernatant was sequentially filtered through a 0.45 μm vacuum filter (VWR, USA) and 0.22 μm bottle-top filter (Corning, USA). The filtrate was pelleted by ultracentrifugation in a 45 TI rotor (Optima XPN-100 Beckman Coulter, USA) at 140,000 ×*g* for 2 h at 4°C. Then, the pellets were re-suspended in phosphate-buffered saline (PBS) as an intact EV suspension and stored at -80°C until use. The total protein concentration of the EVs was measured using a BCA protein assay kit following the manufacturer’s protocol (Thermo Fisher Scientific, USA).

### Animal Experiment and Dosage Information

Three-week-old male senescence-accelerated-resistant (SAMR1) and SAMP8 mice were purchased from Japan SLC and housed under a 12/12 h light/dark cycle. After 6 months of aging induction, the mice were divided into three groups: SAMR1 control group (R1, *n* = 6), SAMP8 control group (P8, *n* = 6), and SAMP8 + *A. muciniphila*-treated group (P8 + *Akk*, *n* = 5). R1 and P8 groups received sterile PBS (containing 0.5 g/l L-cysteine and 20% v/v glycerol) as a vehicle, while the P8 + *Akk* group received live *A. muciniphila* at a concentration of 1 × 10^8^ CFU per mouse. The bacterial dose was selected based on a previous study demonstrating its efficacy in improving muscle function in an immobilization-induced muscular atrophy mouse model [26]. The bacteria were orally administered five times per week for 3 months. Body weight was measured weekly. At the end of the experiment, mice were anesthetized with 2% isoflurane and sacrificed.

All *in vivo* studies were conducted in accordance with institutional and national guidelines. The study protocol was approved by the Korea Food Research Institute Animal Care and Use Committee (KFRI-M-20031).

### Grip Strength Measurement

Grip strength was measured a week before sacrifice using a GT3 test machine (Bioseb, France), as described previously [27]. Five consecutive trials were performed at one-minute intervals, and the average values were used to represent the muscle grip strength of individual mice. The experimenter was blinded to the treatment status of the mice.

### Morris-Water Maze Test

Morris water maze test was performed three weeks before sacrifice, as previously described [28]. Briefly, the mice were subjected to four trials at different starting positions each day for 5 days, and the escape latency was recorded. The probe trial was conducted on day 6 without an escape platform for the spatial memory retention test. All trials were recorded, and the swimming speed was calculated using SMART video-tracking software (Panlab, Spain).

### Histological Analysis

Mouse gastrocnemius muscle was fixed in 4% formaldehyde and embedded in paraffin. The samples were cut into 4-μm sections, deparaffinized, rehydrated, and stained with H&E solution. The mouse colon was also fixed, sectioned, deparaffinized, rehydrated, and stained with H&E and Alcian blue solution. Images of the stained sections were captured using an Olympus BX51 microscope (Olympus Co., Japan). Colon crypt length and mean stained intensity of the colon were quantified using ImageJ software (USA).

### Senescence Associated β- Galactosidase Activity (SA-β-Gal)

SA-β-gal activity was measured using a cellular senescence assay kit (CBA-231; Cell Biolabs, USA) according to the manufacturer’s instructions.

### mtDNA/nDNA Analysis

Relative copy numbers of mitochondrial (mtDNA) and nuclear DNA (nDNA) were analyzed by comparison of 16S rRNA expression relative to hexokinase 2 (*HK2*) expression as described in a previous study [29].

### RT-qPCR Analysis

RNA was extracted using an RNeasy Fibrous Tissue Kit (74704; Qiagen, Germany). cDNA was obtained using qPCR RT Master Mix (FSQ-201; Toyobo, Japan) and a C1000 Touch Thermal Cycler (Bio-Rad Laboratories). A ViiA 7 real-time PCR system (Thermo Fisher Scientific) was used with SYBR Green Master Mix (QPK-201; Toyobo). The mRNA expression levels were analyzed using the 2^−ΔΔCt^ method. The primers used are shown in Table S1.

### Immunoblot

Proteins were extracted using cell lysis buffer and RIPA buffer (89901; Thermo Fisher Scientific) containing protease and phosphatase inhibitors (78440; Thermo Fisher Scientific). After centrifugation, the supernatant was collected. The extracted proteins were quantified and separated using SDS-PAGE . Proteins were transferred to a polyvinylidene fluoride membrane and blocked with 5% skim milk for 1 h. The membrane was incubated with primary antibodies (Table S2) overnight at 4°C. The following day, the membranes were incubated with secondary antibodies for 1 h. Protein expression was detected using G: BOX Chemi XX6 (Syngene, USA) with an ECL western substrate (32106; Thermo Fisher Scientific).

### Total ATP Contents

Total ATP content in skeletal muscle (gastro) was measured using an ATP luminescence detection assay system (ATPlite, PerkinElmer, USA) according to the manufacturer’s instructions.

### Fecal Microbial DNA Extraction and Sequencing

Fresh fecal samples were collected before sacrifice and stored at -80°C until use. DNA was extracted using a DNeasy PowerSoil Kit (Qiagen) according to the manufacturer’s instructions. The extracted DNA was quantified using Quant-IT PicoGreen (Invitrogen, USA). Sequencing libraries were prepared according to the Illumina 16S Metagenomic Sequencing Library protocols to amplify the V3 and V4 regions. Sequencing was performed by Macrogen using the MiSeq platform (Illumina, USA). QIIME (v1.9.0) was used for downstream ASV analysis. Microbial diversity and evenness were calculated using the Shannon and Chao indices. Genetic relationships among samples were visualized based on PCoA.

### Serum Cytokine Analysis

Serum MCP1 (432704) and TNF-α (432704) levels were analyzed using enzyme-linked immunosorbent assay kits purchased from BioLegend (USA) according to the manufacturer’s instructions.

### Cell Culture and Differentiation

C2C12 cells were purchased from ATCC (CRL-1772) and cultured in high glucose Dulbecco’s Modified Eagle Medium (DMEM; SH30243.01; Hyclone, USA) containing 10% fetal bovine serum (FBS; SH30919.03; Hyclone) and penicillin-streptomycin (PS; 15140122; Thermo Fisher Scientific). For myogenic differentiation, C2C12 cells were seeded in 6-well plates and incubated until confluence. The medium was then replaced with a differentiation medium (high-glucose DMEM containing 2% horse serum [26050088, Thermo Fisher Scientific] and 1% PS). The fusion index was calculated as the percentage of nuclei within the myotube per total number of nuclei. To induce myotube atrophy, differentiated C2C12 cells were treated with dexamethasone (5 μM, D4902; Sigma-Aldrich, USA) and *Akk*-EVs (0–10 μg/ml) for 24 h. The diameters of the myotubes were measured using ImageJ software.

### Cell Viability

C2C12 cells were seeded at 1 × 10^4^ cells/well in 96-well culture plates. After 24 h, the cells were treated with varying concentrations (0, 0.625, 1.125, 2.5, 5, and 10 μg/ml) of *Akk*-EVs for 24 h. MTT reagent (5 mg/ml in PBS) was filtered using a 0.45-μm filter. Then, 1/10 volume of the filtered solution was added to the cell culture medium. After 2 h of incubation, the medium was replaced with dimethyl sulfoxide, and the absorbance was measured at 570 nm.

### Immunofluorescence and Myotube Diameter Measurement

Differentiated C2C12 cells were fixed with 4% formaldehyde for 15 min and permeabilized with saponin for 30 min. The cells were blocked with 5% BSA for 1 h at room temperature, followed by incubation with anti-MHC (1:200; MF20, DSHB, USA) at 4°C. After 24 h, the cells were washed thrice with PBS and incubated with Alexa Fluor 488 conjugated secondary antibody (1:500, 4408S; Cell Signaling Technology) for 1 h at room temperature. After washing, the cells were stained with DAPI (10236276001, Sigma-Aldrich). Images were captured using a fluorescence microscope (IX71, Olympus Co.).

### Surface Sensing of Translation (SUnSET) Assay

SUnSET assay was performed as described previously with slight modifications [30]. Briefly, C2C12 cells were differentiated for 4 days, and myotubes were treated with *Akk*-EVs (0–10 μg/ml) and puromycin (1 μg/ml) for 24 h. The incorporation of puromycin was analyzed by immunoblotting using a puromycin antibody.

### Statistical Analysis

Data are presented as the mean ± standard deviation (SD) or the mean ± standard error of the mean (SEM). Significant differences among groups were analyzed using one-way ANOVA followed by Tukey’s post hoc multiple comparison test using GraphPad Prism version 9 (USA). PCoA and PERMANOVA were used to visualize and statistically assess the differences in beta diversity among the groups.

## Results

### *A. muciniphila* Increased Skeletal Muscle Mass and Function

**Fig. 1:**
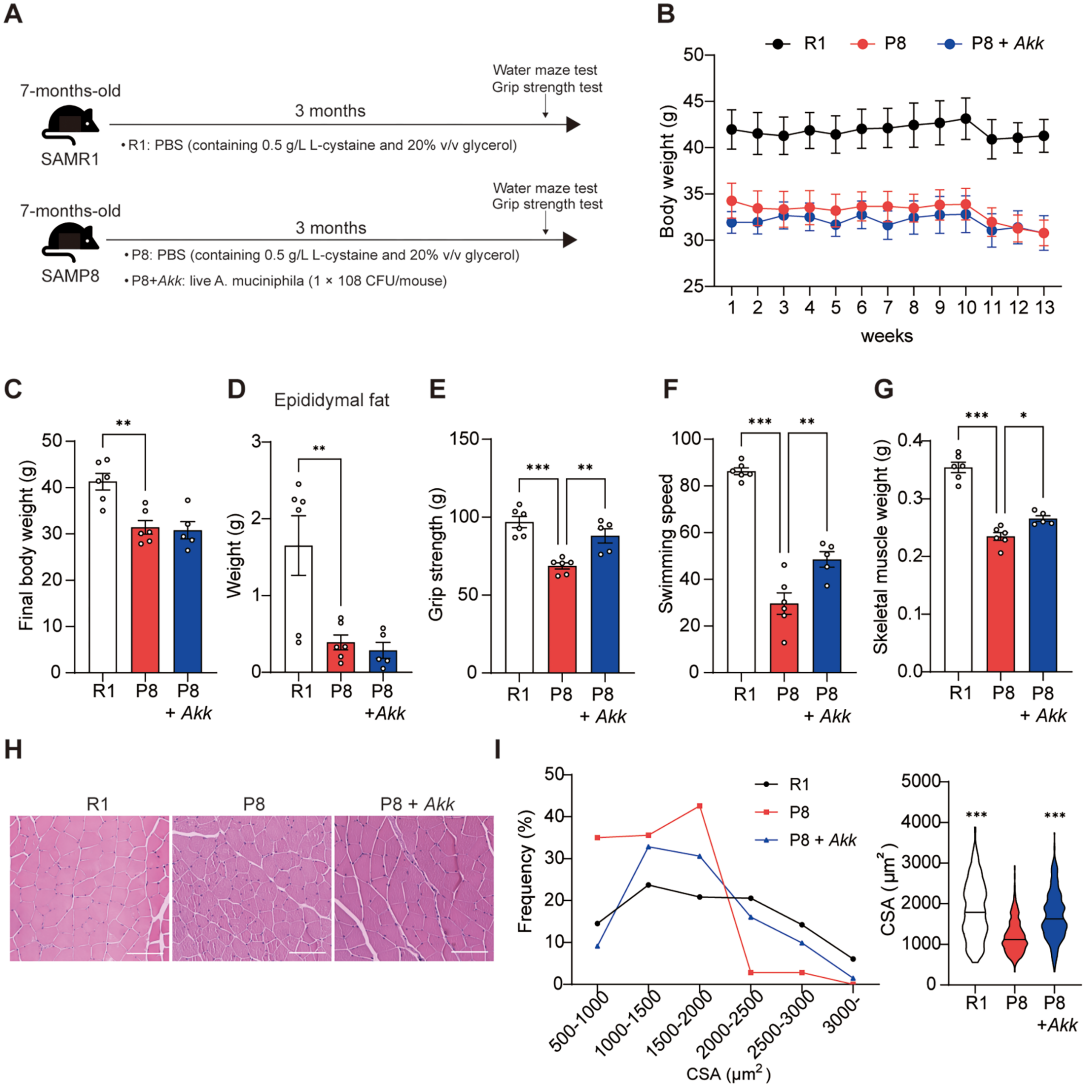
*A. muciniphila* reduced skeletal muscle loss and enhanced exercise capacity in SAMP8 mice. (**A**) Schematic representation of the experimental design. (**B**) Body weight change, (**C**) final body weight, and (**D**) epididymal fat mass in each group. (**E**) Grip strength. (**F**) Swimming speed. (**G**) Skeletal muscle (gastrocnemius) weight. (**H**) Representative images of the gastrocnemius cross-section area (CSA; scale bar = 100 μm). (**I**) Frequency of CSA fiber and average of CSA muscle fiber in the gastrocnemius muscle (n > 300). Data are presented as the mean ± SEM. *p < 0.05, **p < 0.01, ***p < 0.001 vs. P8 group.

To investigate the effect of *A. muciniphila* on sarcopenia, we orally administered *A. muciniphila* to 7-month-old SAMP8 mice for 3 months (Fig. 1A). The final body weight and epididymal fat mass were significantly reduced in SAMP8 mice compared to SAMR1 controls. However, administration of *A. muciniphila* did not result in significant change in these parameters in SAMP8 mice (Fig. 1B–1D). Investigation of the effects of *A. muciniphila* on muscle function through a grip strength test, showed that *A. muciniphila* treatment increased grip strength in SAMP8 mice (Fig. 1E). We also conducted a Morris-water maze test to evaluate the effect of *A. muciniphila* on memory and learning capacity, which revealed no difference between the P8 and P8 + *Akk* groups in the probe trial, whereas *A. muciniphila* improved escape latency in platform trials (Fig. S1A–S1C). *A. muciniphila* also significantly enhanced swimming speed in SAMP8 mice suggesting that *A. muciniphila* mitigated age-related loss of skeletal muscle function (Fig. 1F). Furthermore, assessment of the effects of *A. muciniphila* on skeletal muscle mass revealed significantly increased gastrocnemius weight in the P8 + *Akk* group compared with that in the P8 group (Fig. 1G). *A. muciniphila* administration also significantly increased the muscle fiber size in the P8 + *Akk* group compared with that in the P8 group (Fig. 1H and 1I). These findings suggest that *A. muciniphila* alleviates age-related loss of skeletal muscle mass and function in SAMP8 mice.

### *A. muciniphila* Alleviated Cellular Senescence and Age-Related Skeletal Muscle Atrophy

**Fig. 2:**
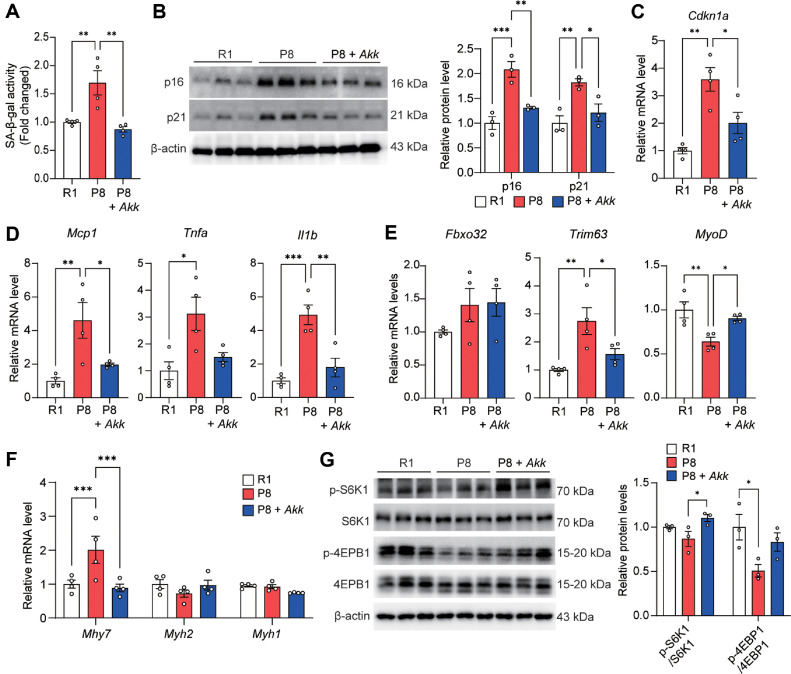
*A. muciniphila* alleviated cellular senescence and age-related skeletal muscle atrophy in SAMP8 mice. (**A**) Quantitative analysis of SA-β-gal activity. (**B**) Protein expression of p16 and p21 and their relative protein levels. (**C–F**) Relative mRNA levels of (**C**) Cdkn1A, (**D**) *Mcp1*, *Tnfa*, and *Il1b*, (**E**) *Fbxo32*, *Trim63*, and *MyoD*, (**F**) *Myh7*, *Myh2*, and *Myh4*. (**G**) Protein expression of p-S6K1, S6K1, p-4EBP1, and 4EBP1 and relative protein levels in gastrocnemius muscle. Data are presented as mean ± SEM. *p < 0.05, **p < 0.01, ***p < 0.001 vs. P8 group.

Next, we assessed the effects of *A. muciniphila* supplementation on cellular senescence in the skeletal muscle to understand the underlying mechanism of its action. The findings showed significantly suppressed SA-β-gal activity in the P8 + *Akk* group compared with that in the P8 group (Fig. 2A). *A. muciniphila* supplementation decreased the protein expression of cellular senescence markers, p21 and p16 in skeletal muscle (Fig. 2B). *A. muciniphila* also reduced the expression of *Cdkn1a* (Fig. 2C) and that of those associated with the senescence-associated secretory phenotype, including *Mcp1*, *Tnfa*, and *Il1b* (Fig. 2D). Furthermore, the expression of *Trim63*, one of the major E3 ubiquitin ligases involved in muscle atrophy within the ubiquitin-proteasome system was significantly reduced in the P8 + *Akk* group compared with that in the P8 control group. In contrast, *A. muciniphila* supplementation increased the expression of the myogenic regulatory factor *MyoD* (Fig. 2E). The composition of muscle fiber type can change due to various stressors, and during aging, the composition of muscle fibers shifts with relative increase in type 1 and decrease in type 2 muscle fibers [31]. Our findings showed that the mRNA level of *Myh7* was lower in the P8 + *Akk* group than that in the P8 group. In contrast, the mRNA level of *Myh2* tended to increase in the P8 + *Akk* group compared with that in the P8 group, suggesting that *A. muciniphila* delayed the changes in age-related muscle fiber type composition (Fig. 2F). The mTORC1 signaling pathway is a major protein synthesis pathway, in which mTORC1 activates the protein synthesis pathway by phosphorylating S6K1 and 4EBP1 [32]. In the present study, we observed increased phosphorylation of both proteins in the P8 + *Akk* group compared with that in the P8 group (Fig. 2G). These findings suggest that *A. muciniphila* alleviates sarcopenia by suppressing cellular senescence and improving the balance between protein synthesis and degradation.

### *A. muciniphila* Improved Mitochondrial Function

**Fig. 3:**
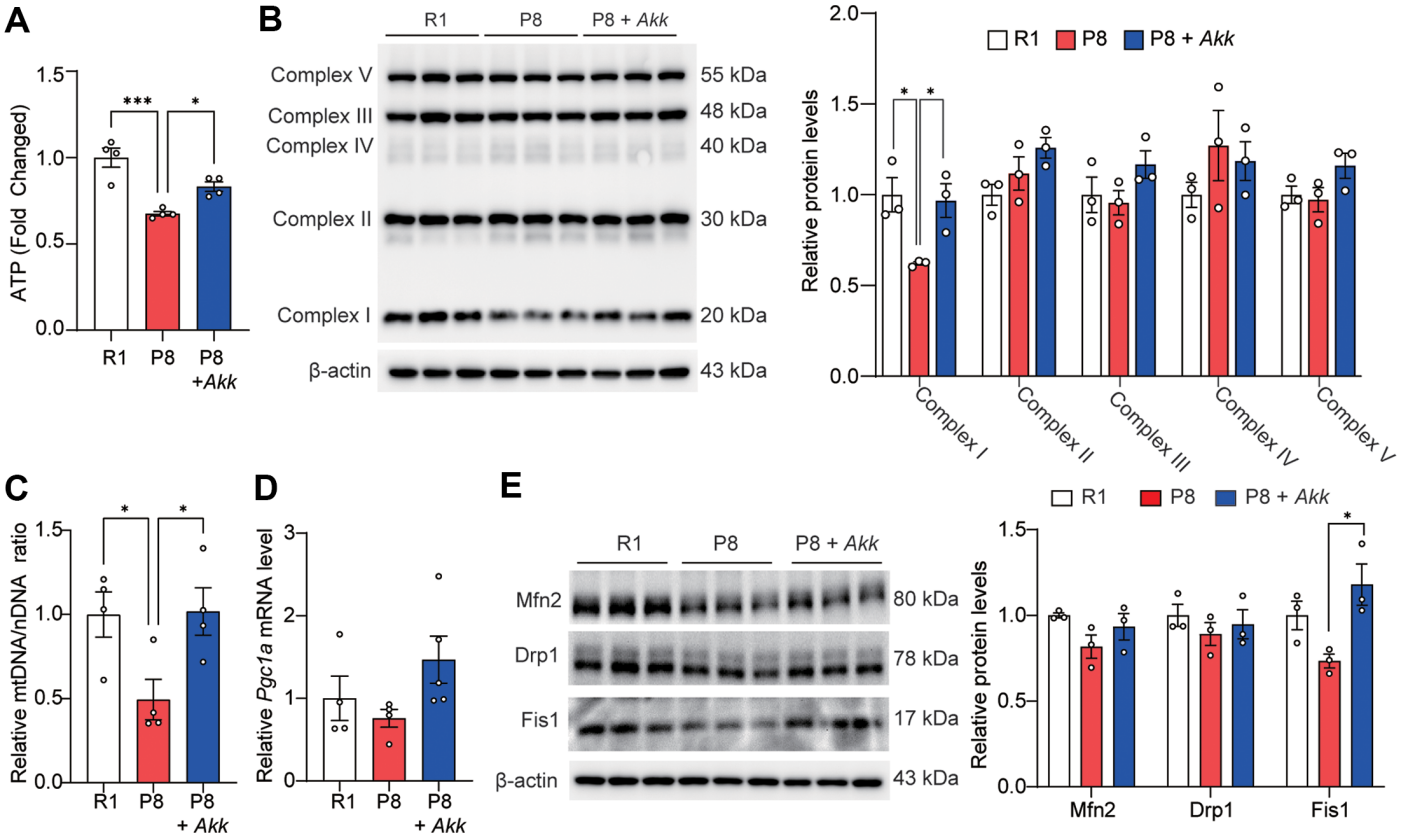
*A. muciniphila* enhances mitochondrial function in SAMP8 mice. (**A**) Relative total ATP levels (*n* = 4 per group). (**B**) Protein expression of OXPHOS complexes and relative protein levels. (**C**) Relative mtDNA/nDNA ratios. (**D**) Relative mRNA expression of *Pgc1a*. (**E**) Expression of Mfn2, Drp1, and Fis1 proteins and their relative levels in the gastrocnemius muscle. Data are presented as mean ± SEM. *p < 0.05, **p < 0.01, ***p < 0.001 vs. P8 group.

Next, we investigated the effects of *A. muciniphila* on mitochondrial function involved in skeletal muscle function by measuring the total ATP content and the expression of the mitochondrial OXPHOS complex. The total ATP content in the gastrocnemius (Fig. 3A) and the expression of OXPHOS complex 1 was higher in the P8 + *Akk* group than those in the P8 control group (Figs. 3B and S2). To further analyze the effect of *A. muciniphila* on mitochondrial function, we investigated its effects on mitochondrial biogenesis. The findings demonstrated increased mtDNA and upregulated *Pgc1a*, a master regulator of mitochondrial biogenesis, in the P8 + *Akk* group compared with that in the P8 group (Fig. 3C and 3D). Mitochondrial dynamics, including fusion, fission, and degradation, are regulated by various factors and are crucial for mitochondrial function [33]. In the present study, the expression of mitochondrial fusion and fission-related proteins, including Mfn2, Drp1, and Fis1, was higher in the P8 + *Akk* group than that in the P8 group (Fig. 3E). These findings suggest that *A. muciniphila* alleviates mitochondrial dysfunction in SAMP8 mice.

### *A. muciniphila* Supplementation Modified Gut Microbiota Community

**Fig. 4:**
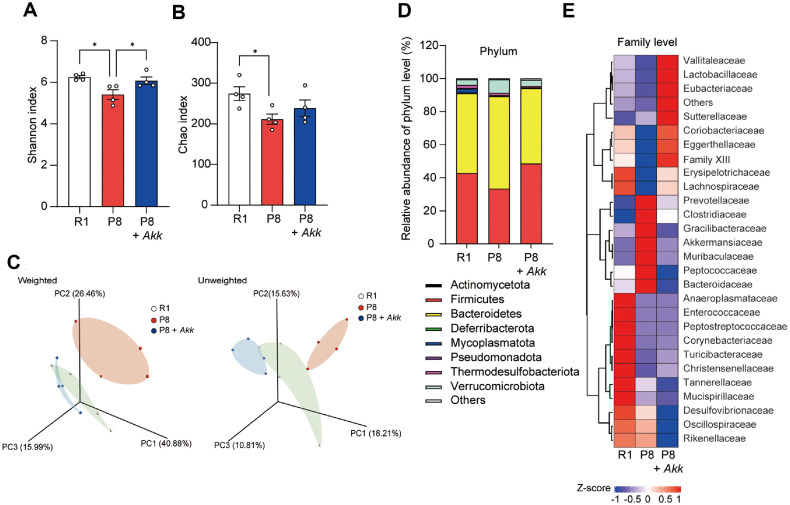
*A. muciniphila* supplementation modifies the gut microbiota composition. 16S rRNA sequencing was performed on mouse feces (*n* = 4 per group), and diversity was assessed using (**A**) Shannon index, (**B**) Chao index, and (**C**) principal coordinate analysis of the weighted (left) and unweighted (right) UniFrac. (**D**) Relative abundance at the phylum level. (**E**) Heatmap of the microbial composition at the family level. Data are presented as mean ± SEM. *p < 0.05, **p < 0.01, ***p < 0.001 vs. P8 group.

Next, we investigated the effects of *A. muciniphila* supplementation on the gut microbiota of SAMP8 mice using 16S rRNA sequencing. The Shannon and Chao indices revealed lower alpha diversity in P8 group than that in SAMR1 (R1) group, which was reversed by *A. muciniphila* supplementation in P8 + *Akk* group (Fig. 4A and 4B). Beta diversity analysis using principal coordinate analysis (PCoA) showed distinct clustering of the R1 and P8 groups, with the microbiota composition in the P8 + *Akk* group shifting closer to that of the R1 group (Figs. 4C, S3A, and S3B). Permutational multivariate ANOVA (PERMANOVA) analysis based on weighted UniFrac distances revealed no significant differences in beta diversity among groups (*p* = 0.062, R² = 0.295). In contrast, unweighted UniFrac-based analysis showed a significant difference (*p* = 0.002, R² = 0.2705, Table S3), suggesting that low-abundance microbial taxa contributed to the observed distinctions between the groups. These findings suggest that *A. muciniphila* supplementation improves gut microbial diversity in SAMP8 mice.

Analysis of the gut microbiota composition identified *Firmicutes* and *Bacteroidetes* as the most prevalent bacterial phyla, accounting for 90% of the sequences on average (Fig. 4D). Furthermore, the relative abundance of *Bacteroidetes* increased, and that of *Firmicutes* decreased to some degree in SAMP8 mice compared with those in SAMR1 mice. The *Firmicutes*/*Bacteroidetes* (F/B) ratio is an indicator of gut dysbiosis, and alterations in this ratio are associated with the development of certain diseases, including inflammatory bowel disease and obesity [34]. *A. muciniphila* supplementation altered the F/B ratio in a similar trend as in R1 group, suggesting that *A. muciniphila* alleviated gut microbial imbalance in SAMP8 mice (Fig. S4A). The heatmap of relative abundance at the family level showed clear separation among groups (Fig. 4E). Our findings confirmed that *A. muciniphila* supplementation significantly increased the abundance of *Lactobacillaceae* (*p* < 0.001), *Lachnospiraceae* (*p* = 0.005), *Eggerthellaceae* (*p* = 0.008), and *Eubacteriales* Family XIII (*p* = 0.009) in the P8 + *Akk* group compared with that in the P8 group (Fig. S4B). Particularly at the genus level, *Limosilactobacillus* (*p* = 0.043), *Blautia* (*p* = 0.062), *Coprococcus* (*p* = 0.025), *Jutongia* (*p* = 0.012), *Sporofaciens* (*p* = 0.031), *Berryella* (*p* = 0.019), *Inhubacter* (*p* = 0.003), and *Zhenpiania* (*p* = 0.01) were significantly increased by *A. muciniphila* supplementation (Fig. S4C). These findings indicate that *A. muciniphila* supplementation enhances gut microbial diversity and alleviates gut microbial imbalance.

### *A. muciniphila* Enhanced Tight Junction Integrity and Reduced Systemic Inflammation

**Fig. 5:**
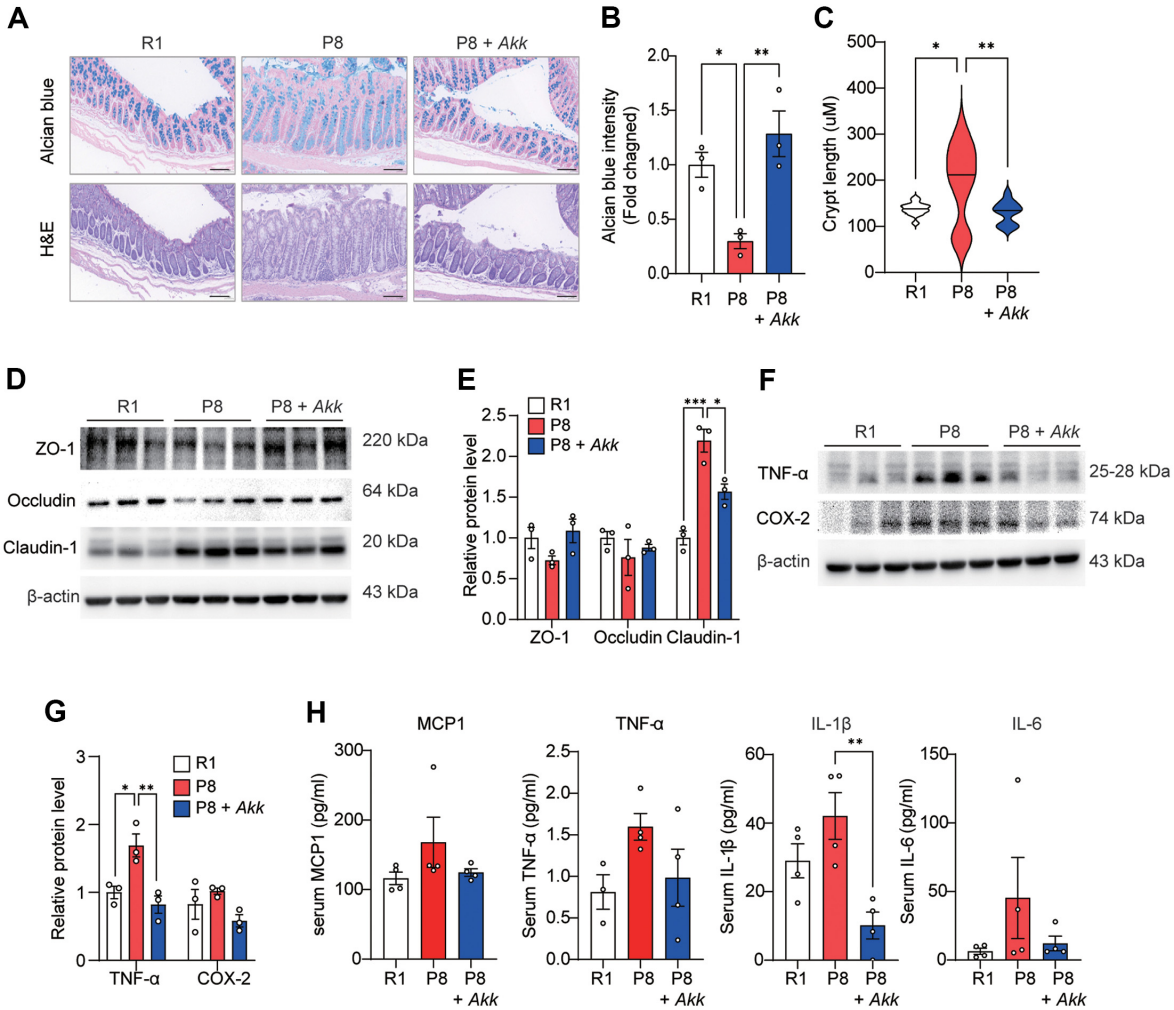
*A. muciniphila* enhances tight junction integrity and reduces systemic inflammation. (**A**) Representative image of colon tissue stained with alcian blue (top) and H&E (bottom) from each group (scale bar = 100 μm). (B, C) Quantitative analysis of (**B**) alcian blue staining and (**C**) crypt length. (**D**) Expression of tight junction proteins ZO-1, Occludin, and Claudin-1 and (**E**) relative protein levels. (**F**) Expression of TNF-α and COX-2 and (**G**) their relative levels in the mouse colon. (**F**) Serum MCP1, TNF-α, IL-1β, and IL-6 levels. Data are presented as mean ± SEM. *p < 0.05, **p < 0.01, ***p < 0.001 vs. P8 group.

Histological evaluation of intestinal inflammation assessed using Alcian blue and H&E staining revealed that *A. muciniphila* treatment alleviated intestinal inflammation levels in the treated group (Fig. 5A). The mean staining intensity of mucin was lower in the P8 group than that in the R1 group, which was reversed by *A. muciniphila* supplementation in the P8 + *Akk* group (Fig. 5B). A previous study reported that crypt length increases depending on the severity of inflammation [35], and we observed that the increased crypt length in SAMP8 mice was reduced by *A. muciniphila* treatment (Fig. 5C), suggesting that *A. muciniphila* supplementation reduced mucin degradation and mitigated inflammation in the colon.

Loss of tight junction integrity increases intestinal permeability, leading to infections and widespread inflammation [36]. The expression levels of ZO-1 and Occludin, which contribute to tight junction barrier function, were markedly reduced in the colon of SAMP8 mice compared with those in SAMR1 mice but increased in *A. muciniphila*-treated mice. However, the expression of Claudin-1, which is related to inflammatory bowel diseases [37], was reduced in the P8 + *Akk* group (Fig. 5D and 5E). In addition, the expression of two major inflammatory markers, TNF-α and COX-2, was reduced in the P8 + *Akk* group compared with that in the P8 group (Fig. 5F and 5G). Consistently, *A. muciniphila* supplementation significantly reduced serum IL-1β and tended to reduce serum MCP-1, TNF-α, and IL-6 levels in SAMP8 mice (Fig. 5H). These findings suggest that *A. muciniphila* inhibits intestinal and systemic inflammation by enhancing tight junction integrity in SAMP8 mice.

### *Akk*-EVs Promoted Myogenesis and Suppressed Dexamethasone-Induced Muscle Atrophy in C2C12 Myoblast

**Fig. 6:**
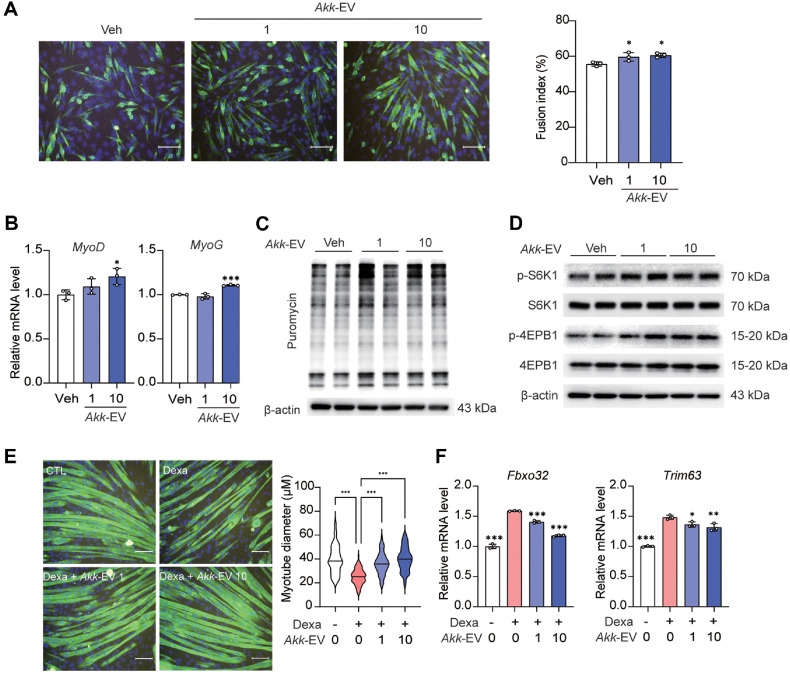
Effect of *A. muciniphila*-derived extracellular vesicle on C2C12 myoblast differentiation and muscle atrophy. (**A**) Representative image of differentiated C2C12 cells treated with *Akk*-EVs (0–10 μg/ml) and quantitative analysis of the fusion index (scale bar = 200 μm). (**B**) Relative mRNA expression of myogenic-related genes, *MyoD* and *MyoG*, in differentiated C2C12 cells treated with *Akk*-EVs (0–10 μg/ml). (**C**) Relative protein synthesis level in differentiated C2C12 cells treated with *Akk*-EVs (0–10 μg/ml) analyzed using the SUnSET assay. (**D**) Expression levels of p-S6K1, S6K1, p-4EBP1, and 4EBP1 in differentiated C2C12 cells treated with *Akk*-EVs (0–10 μg/ml). (**E**) Representative images of myotubes treated with dexamethasone and *Akk*-EVs (0–10 μg/ml) and quantitative analysis of myotube diameter (scale bar = 200 μm). (**F**) Relative mRNA expression of *Fbxo32* and *Trim63* in myotubes treated with dexamethasone and *Akk*-EVs (0–10 μg/ml). Data are presented as mean ± SD. *p < 0.05, **p < 0.01, ***p < 0.001 vs. control.

*Akk*-EVs play a bioactive role by interacting with the gut microbiota and host [22]. Nano-sized bacterial extracellular vesicles carry various cargo and enter the bloodstream, accessing peripheral tissue and regulating various cellular and molecular pathways [38]. Therefore, we hypothesized that *Akk*-EVs contribute to enhanced skeletal muscle mass. We isolated *Akk*-EVs and analyzed their morphology and size of *Akk*-EVs using transmission electron microscopy (TEM) and dynamic light scattering analysis (Fig. S5A and S5B). TEM imaging analysis showed that *Akk*-EVs were spherical and had lipid bilayers, and the size of *Akk*-EVs measured by DLS was consistent with a previous study using *Akk*-EVs [22]. To test this hypothesis, we examined whether *Akk*-EVs enhance myogenic differentiation within non-toxic doses of *Akk*-EVs on cell viability of C2C12 (Fig. S5C). We treated C2C12 cells with *Akk*-EVs and induced their differentiation. *Akk*-EVs increased the fusion index and number of myotubes in C2C12 cells (Fig. 6A). Additionally, the expression of myogenesis-related genes was upregulated by *Akk*-EVs treatment (Fig. 6B), suggesting that *Akk*-EVs enhanced myogenesis. Furthermore, protein synthesis measured using the SUnSET assay, was increased in *Akk*-EVs-treated cells (Fig. 6C). *Akk*-EVs also enhanced the phosphorylation of S6K1 and 4EBP1 (Fig. 6D). To determine whether this effect was specific to *Akk*-EVs or also observed in heat-inactivated *A. muciniphila*, we treated the cells with heat-inactivated *A. muciniphila* and induced differentiation. No significant effects were observed in the heat-inactivated *A. muciniphila*-treated C2C12 cells (Fig. S6A–S6D). Next, we investigated the effect of *A. muciniphila* on dexamethasone-induced muscle atrophy in C2C12 cells. *Akk*-EVs treatment dose-dependently increased myotube diameter in dexamethasone-treated cells (Fig. 6E). In addition, the *Akk*-EVs treatment suppressed the mRNA expression of *Fbxo32* and *Trim63* (Fig. 6F). Together, these findings suggest that *Akk*-EVs enhance myogenesis and suppress myotube atrophy.

## Discussion

Emerging research has highlighted a strong relationship between gut microbiota and muscle health. A previous study demonstrated that *A. muciniphila* is positively associated with healthy muscle mass and composition [39]. For instance, in an immobilization-induced muscular atrophy mouse model, supplementation with *A. muciniphila* improved muscle strength, while in a streptozotocin-induced muscle atrophy model, it mitigated muscle loss and restored muscle functionality [26, 40]. These findings suggest that *A. muciniphila* may exert protective effects on muscles under stress or pathological conditions. Building on these foundational studies, we focused on the role of *A. muciniphila* in a SAMP8 mouse model. SAMP8 mice are one of the SAMP substrains obtained through phenotypic selection from a common genetic pool of AKR/J strain mice, which includes nine major SAMP substrains and three SAMR mice [41]. SAMP8 mice are commonly used as a mouse model of age-related cognitive impairment and aging-related muscle atrophy [42, 43]. A previous study demonstrated that age-related muscle loss in SAMP8 mice progresses twice as fast as that in SAMR1 and C57BL/6 mice [44]. In addition, age-related changes in gut microbiota have been observed in SAMP8 mice starting at 6 months of age [45]. Unlike the induced atrophy models used in previous studies, the SAMP8 model provides an opportunity to study the effects of *A. muciniphila* in a natural aging system, reflecting more physiologically relevant conditions. This distinction allowed us to evaluate its efficacy in addressing muscle loss driven by aging processes and understand its interplay with the gut–muscle axis, particularly in modulating systemic inflammation and metabolic pathways contributing to sarcopenia.

We investigated whether the effect of *A. muciniphila* could be attributed to an improvement in the gut microbiota community in SAMP8 mice. *A. muciniphila* supplementation increased the abundance of *Lactobacillaceae*, *Lachnospiraceae*, *Eubacteriales* Family XIII, and *Eggerthellaceae* at the family level in SAMP8 mice. *Lactobacillaceae* and *Lachnospiraceae* can produce SCFAs, such as butyrate and lactic acid, which exhibit anti-inflammatory and immunomodulatory effects [46-48]. Previous studies have reported that butyrate alleviates muscle atrophy and enhances mTORC1 signaling. Other SCFAs, such as acetate and propionate, are also related to skeletal muscle function [49, 50]. At the genus level, the abundance of SCFA-producing bacteria *Blautia* has been shown to be increased by berberine and metformin treatment in a high-fat diet-induced rat model and shown to improve gastrointestinal health. Similarly, *Coprococcus eutactus*, a potent probiotic with SCFA-producing properties, ameliorated colitis [51]. *Limosilactobacillus reuteri ID-D01* has been reported to increase SCFA production, enhance exercise performance, and reduce fatigue in Sprague-Dawley rats [52]. Considering these findings, we speculated that *A. muciniphila* supplementation could increase SCFA production. However, further studies are needed to determine whether the effects of *A. muciniphila* on skeletal muscle mass and function are mediated by metabolites of beneficial bacteria, which are increased in SAMP8 mice. Although *A. muciniphila* was administered orally, 16S rRNA gene-based analysis of fecal microbiota did not reveal a statistically significant change in the relative abundance of the *Akkermansiaceae* family. This discrepancy may be explained by the possibility that the administered strain did not achieve stable colonization but instead exerted transient effects on the gut environment. Previous studies have reported beneficial outcomes such as improved lipid absorption and attenuation of the age-related colonic mucus thinning, even in the absence of sustained colonization [53, 54]. These findings imply that *A. muciniphila* may exert its physiological effects through indirect mechanisms such as modulation of mucosal immune responses, stimulation of SCFA production, or remodeling of the gut microbial community, rather than through long-term engraftment. Further investigations utilizing strain-specific detection techniques or mucosal tissue-based analyses are warranted to elucidate the localization, persistence, and mechanisms of action of *A. muciniphila* within the host.

The gut barrier is crucial in protecting the host against gut microbes, food antigens, and toxins [55]. Gut barrier dysfunction and increased intestinal permeability increase the leakage of toxic bacterial metabolites into circulation, leading to the development of low-grade systemic inflammation [56]. Therefore, we investigated the effects of *A. muciniphila* on gut barrier integrity and inflammation. Our results demonstrated that *A. muciniphila* increased the expression of tight junction proteins and suppressed inflammation in the intestine. These findings are consistent with those of previous studies demonstrating that *A. muciniphila* can mitigate the impairment of the intestinal barrier and suppress inflammation induced by metabolic endotoxemia [57, 58]. In addition, *A. muciniphila* supplementation reduced the expression of inflammatory cytokines in the skeletal muscle. Considering the gut–muscle axis, increased levels of circulating inflammatory cytokines can affect skeletal muscle, potentially leading to skeletal muscle inflammation and aging. These findings suggest that the effects of *A. muciniphila* on gut barrier integrity may alleviate sarcopenia by reducing circulating inflammation.

Our results from C2C12 cells revealed that *Akk*-EVs are a potential contributor to this effect. Bacterial EVs are mediators of inter-bacterial signaling and microbiota-host interactions [59]. Therefore, we speculated that *A. muciniphila* exerts a beneficial effect not only by balancing the gut microbiota, but also by its EVs. Even though orally administered bacteria may not directly adhere to the host intestine, they can still produce EVs, such as exosomes, ectosomes, and apoptotic bodies, which can enter the bloodstream and exert various effects [47]. Previous studies have shown that *Akk*-EVs increase the protein expression of occludin, improve intestinal barrier integrity [22], alleviate obesity, and inhibit liver inflammation [23, 24]. However, their effects on myogenesis and skeletal muscle atrophy have not been fully elucidated. To investigate this, we used an *in vitro* myogenic differentiation model and a dexamethasone-induced muscle atrophy model using C2C12 myoblasts. Dexamethasone, a synthetic glucocorticoid, induces skeletal muscle atrophy by inhibiting protein synthesis and promoting protein degradation [60]. Dexamethasone binds to the glucocorticoid receptor, facilitating its translocation into the nucleus and promoting the transcription of atrophy-related genes, such as *Atrogin-1* and *MuRF1*, via activation of the forkhead transcription factor FoxO3a [61]. Owing to these mechanisms, dexamethasone is commonly used to model muscle atrophy, typically at 1–10 μM *in vitro* and 10–20 mg/kg *in vivo* [62-64]. In our study, we used 5 μM dexamethasone, a concentration within the validated range that has been shown to reliably induce muscle atrophy in C2C12 cells [28]. Our findings demonstrated that *Akk*-EVs enhanced myogenesis and protein synthesis and suppressed dexamethasone-induced muscle atrophy. Together, these findings suggest the potential of *Akk*-EVs as new postbiotic agents for the treatment or prevention of sarcopenia.

While our study provides valuable insights into the effects of *A. muciniphila* and *Akk*-EVs on muscle health, some limitations remain. For instance, we did not perform a detailed proteomic analysis to identify the specific protein constituents of *Akk*-EVs responsible for the observed effects. Future studies using techniques like liquid chromatography-mass spectrometry/mass spectrometry will be crucial in uncovering the molecular pathways regulated by *Akk*-EVs. In vivo studies and pharmacokinetic analyses are necessary to further validate the anti-sarcopenic effects of *Akk*-EVs and establish their therapeutic potential. Another limitation is the lack of germ-free or antibiotic-treated control groups, which restricts our ability to attribute the observed effects exclusively to *A. muciniphila*. Although our findings indicate that *A. muciniphila* plays a role in modulating age-related physiological changes, the contribution of the broader shifts in the overall gut microbiota to the observed outcomes cannot be ruled out. Future studies incorporating germ-free or antibiotic-treated models would help delineate the direct effects of *A. muciniphila* and further validate our findings.

In conclusion, our findings demonstrate that *A. muciniphila* supplementation mitigated the loss of skeletal muscle mass and enhanced skeletal muscle function in SAMP8 mice. This anti-sarcopenic effect of *A. muciniphila* was achieved by enhancing gut microbiota composition, improving gut barrier integrity, and reducing systemic inflammation. In addition, *Akk*-EVs contributed to enhancing myogenesis. These findings suggest that *A. muciniphila* and its EVs may offer a promising new therapeutic option to treat sarcopenia.

## Supplemental Materials

Supplementary data for this paper are available on-line only at http://jmb.or.kr.
