# Supplementary material for: Akkermansia muciniphila Alleviates Sarcopenia in Senescence-Accelerated Mouse-Prone 8 Mice
Source: J Microbiol Biotechnol. 2025 Aug 26;35:e2507001. doi: 10.4014/jmb.2507.07001 (PMC12409435; doi:10.4014/jmb.2507.07001)
Supplement: Supplementary file 1 [file jmb-35-e2507001-supple.pdf]

J. Microbiol. Biotechnol. 2025. 35: e2507001

https://doi.org/10.4014/jmb.2507.07001

Supplementary Figures and Tables

Morris-water maze test

Morris-water maze test was performed three weeks before sacrifice according to previous described [1]. Briefly, The mice were subjected to four trials at different starting positions each day for 5 days, and the escape latency was recorded. The probe trial was conducted on day 6 without an escape platform for the spatial memory retention test. All trails were recorded, and the swimming speed was calculated using SMART video-tracking software (Panlab, Spain).

Cell viability

C2C12 cells were seeded at 10,000 cells per well in 96-well culture plates. After 24 h, the cells were treated with varying concentrations of *Akk*-EVs (0–10 μg/mL) or heat-inactivated *A. muciniphila* (treatment for 30 min at 70°C, 0–1×10^8) for 24 h. MTT reagent (5 mg/mL in PBS) was filtered using a 0.45-μm filter. 1/10 volume of the filtered solution was added to the cell culture medium. After 2 h of incubation, the media were replaced with dimethyl sulfoxide and the absorbance was measured at 570 nm.

Cryo-EM grid preparation and imaging

4 μL of sample was applied onto holey carbon grid (Quantifoil R1.2/1.3 Cu 200 mesh, SPI), which had been negatively glow-discharged at 15 mA for 1 min using an easiGlow (PELCO, USA) device. The grids were then blotted for 5 sec using a Vitrobot Mark IV (Thermo Fisher Scientific Inc., USA) at 4°C and 95% humidity, followed by plunge-freezing in liquid ethane cooled by liquid nitrogen. Cryo-EM images were acquired at the Korea Basic Science Institute

using a Talos Arctica G2 (Thermo Fisher Scientific Inc., USA) transmission electron microscope operated at 200 kV. The microscope was equipped with a K3 direct electron detector (Gatan Inc., USA) and images were captured using low-dose imaging with a total dose of less than 50 e−/Å2.

Dynamic light scattering analysis

AKK-EVs were diluted in PBS to a concentration of 2 μg/mL, and their size was measured using dynamic light scattering (DLS) with a Zetasizer Nano ZS (Malvern Instruments, UK) according to the manufacturer's instructions.

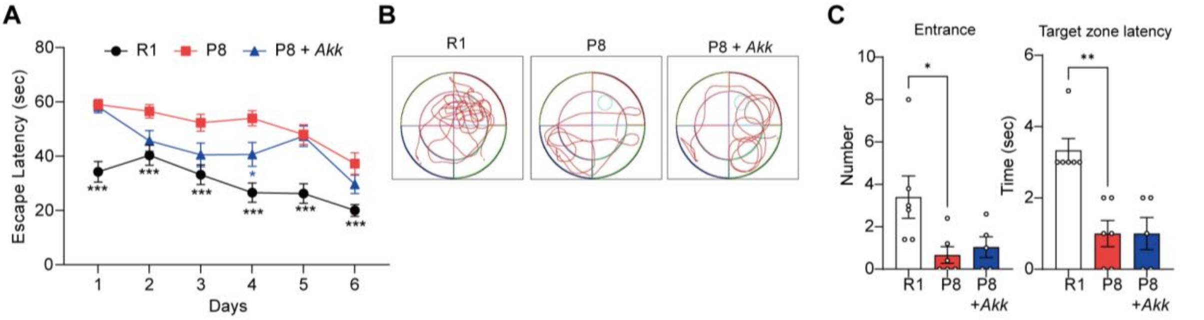

Fig. S1. Effect of *A. muciniphila* supplementation on memory function. (A) Escape latency in each group for six consecutive days. **(B)** Swimming paths of each group on the sixth day. **(C)** Target zone entrance number and latency in the probe trial. Data are presented as the mean ± SEM. *$p < 0.05$, **$p < 0.01$, ***$p < 0.001$ vs. SAMR1 control group.

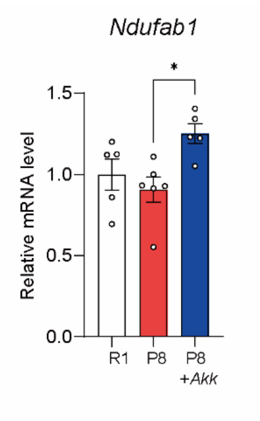

Fig. S2. Relative mRNA levels of *Ndufab1* (n = 4 per group). Data are presented as the mean ± SEM. *$p < 0.05$ vs. SAMR8 control group.

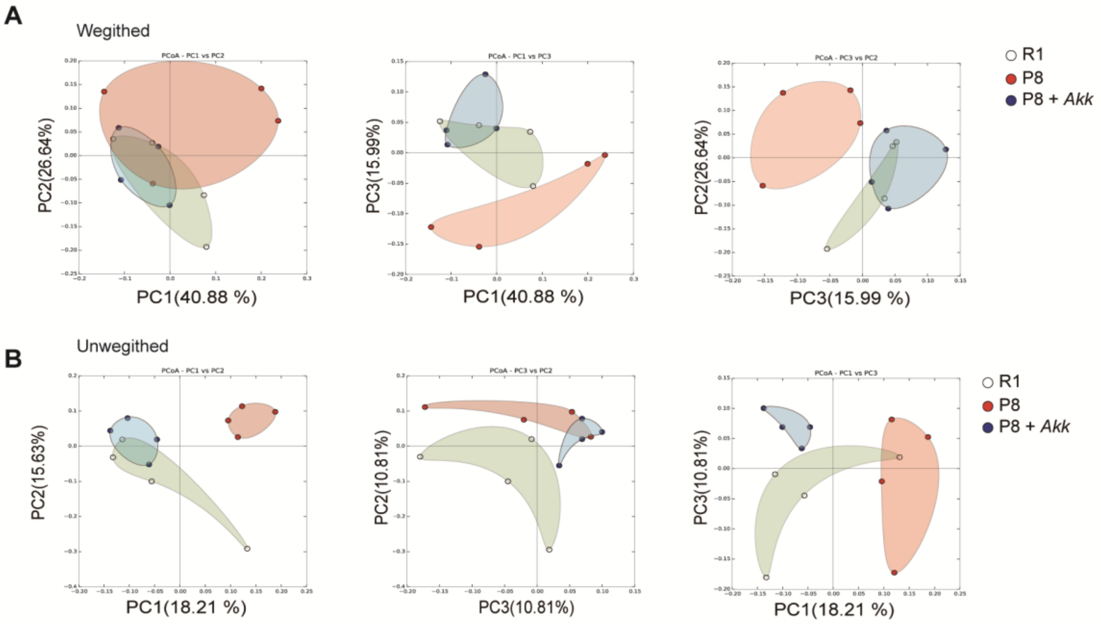

Fig. S3. Effect of AMA on Gut Microbial Community Clustering. Two-dimensional PCoA plots based on **(A)** weighted and **(B)** unweighted UniFrac distances, illustrating clustering patterns among the analyzed groups.

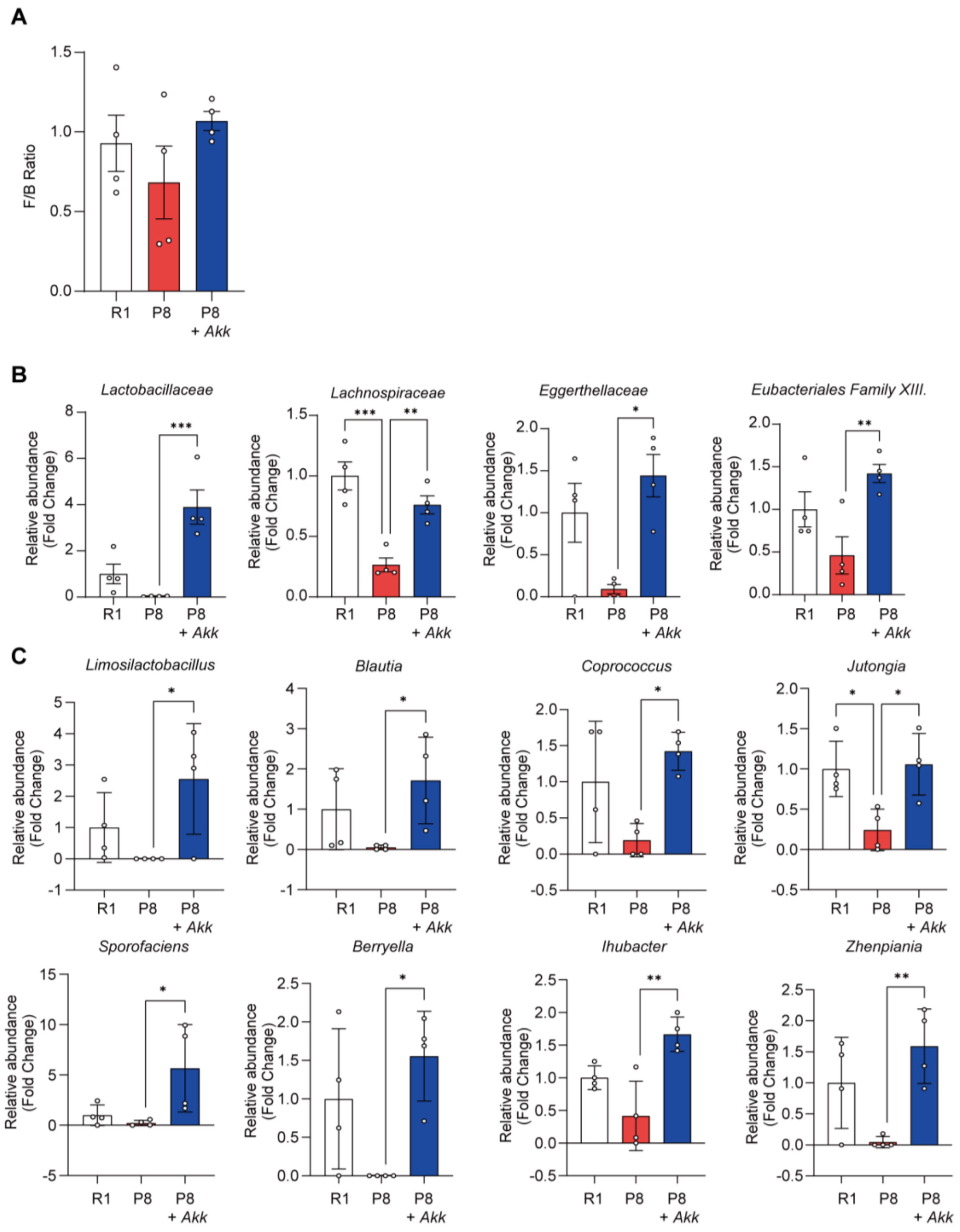

Fig. S4. Effect of *A. muciniphila* on the gut microbiota community. (A) *Firmicutes/Bacteroidetes* (F/B) ratio. **(B)** Relative abundance of *Lactobacillaceae*, *Lachnospiraceae*, *Eggerthellaceae*, and *Eubacteriales family XIII.* Only the family levels with significant differences are presented, and the genus levels within these families that showed significant differences are as follows: **(C)** relative abundance of *Limosilactobacillus*, *Blautia*, *Coprococcus*, *Jutongia*, *Sporofaciens*, *Berryella*, *Inhubacter*, and *Zhenpiania.* Data are presented as mean ± SEM. *$p < 0.05$, **$p < 0.01$, ***$p < 0.001$ vs. SAMR8 control group.

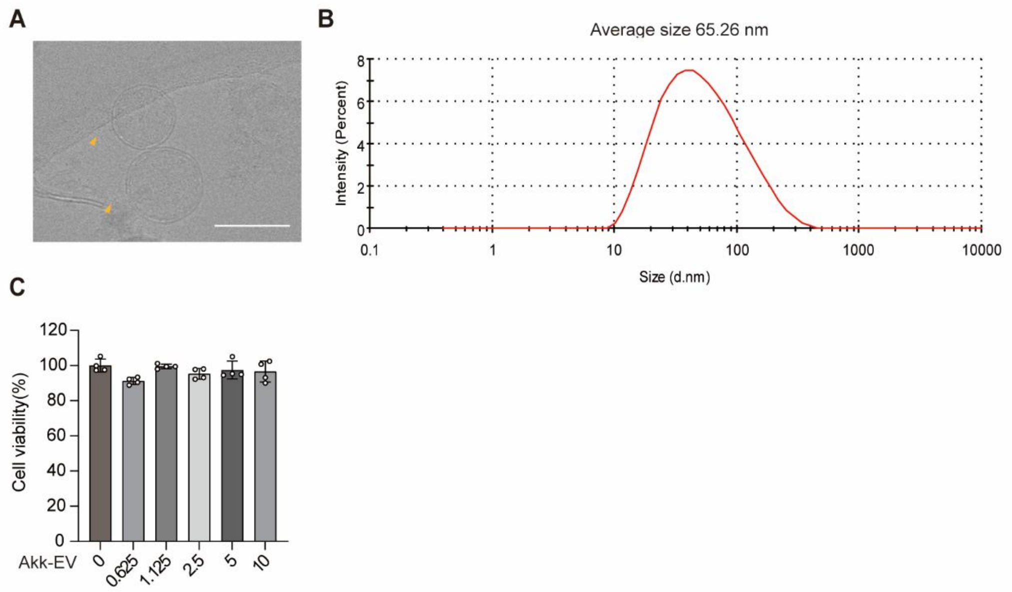

Fig. S5. Characterization of *A. muciniphila*-derived extracellular vesicles (*Akk*-EVs) and the effect of *Akk*-EVs on C2C12 cell viability. (A) Representative transmission electron microscopic (TEM) image of *Akk*-EVs (scale bar = 100 nm). **(B)** *Akk*-EVs size (d.nm) was measured using dynamic light scattering (DLS). (**C)** The effect of *Akk*-EVs on cell viability were measured by the MTT assay. Data are presented as mean ± SD.

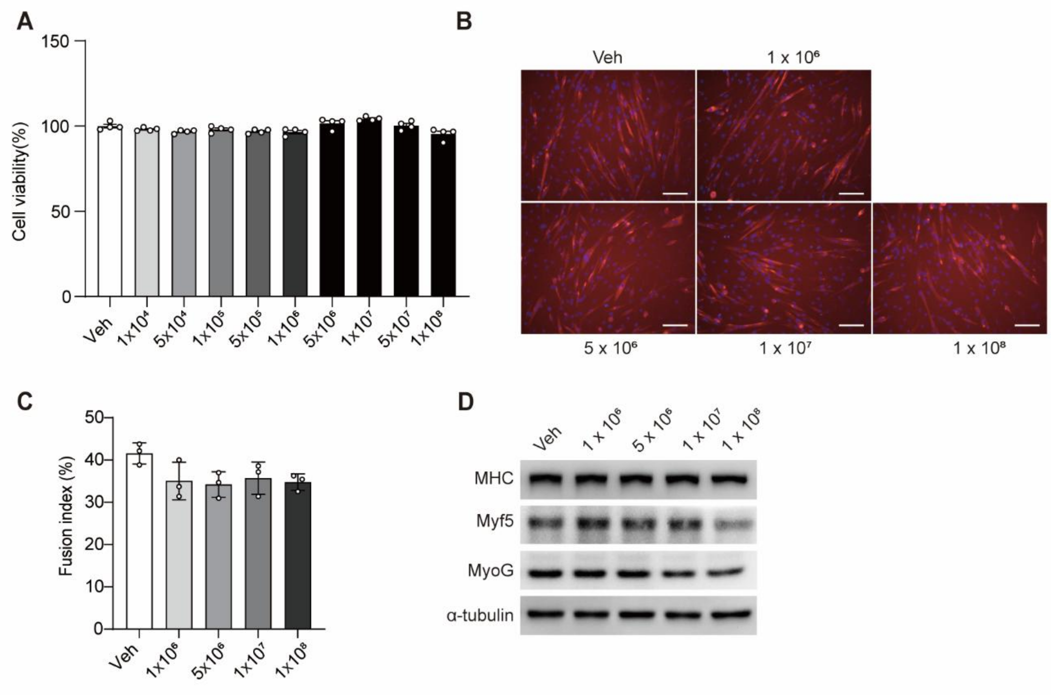

Fig. S6. Effect of heat-inactivated *A. muciniphila* on cell viability and cell differentiation in C2C12 cell. (A) The effect of heat-inactivated *A. muciniphila* on cell viability were measured by the MTT assay. **(B)** Representative image of differentiated C2C12 cells treated with various concentrations ($0–1×10^8$) of heat-inactivated *A. muciniphila* and **(C)** quantitative analysis of the fusion index (scale bar = 200 μm). **(D)** Protein expression levels of MHC, Myf5, and MyoG in differentiated C2C12 cells treated with various concentrations ($0–1×10^8$) of heat-inactivated *A. muciniphila.* Data are presented as mean ± SD.

Table S1. Primer sequences

Genes		Sequences
Atrogin1	Forward	5'- AAGGCTGTTGGAGCTGATAGCA -3'
	Reverse	5'- CACCCACATGTTAATGTTGCCC -3'
MuRF1	Forward	5'- TGTCTCACGTGTGAGGTGCCTA -3'
	Reverse	5'- CACCAGCATGGAGATGCAGTTAC -3'
MyoD	Forward	5'- CCGTGTTTCGACTCACCAGA -3'
	Reverse	5'- GTAGTAGGCGGTGTCGTAGC -3'
MyoG	Forward	5'- ATGGTGCCCAGTGAATGCAAC -3'
	Reverse	5'- CCACGATGGACGTAAGGGAG -3'
Myh7	Forward	5'- CTCAAGCTGCTCAGCAATCTATTT -3'
	Reverse	5'- GGAGCGCAAGTTTGTCATAAGT -3'
Myh2	Forward	5'- AAGCGAAGAGTAAGGCTGTC -3'
	Reverse	5'- GTGATTGCTTGCAAAGGAAC -3'
Myh4	Forward	5'- CACCTGGAGCGGATGAAGAAGAAC-3'
	Reverse	5'- GTCCTGCAGCCTCAGCACGTT -3'
Cdkn1A	Forward	5'-CCAGGCCAAGATGGTGTCTT -3'
	Reverse	5'- TGAGAAAGGATCAGCCATTGC -3'
Mcp1	Forward	5'- CCACTCACCTGCTGCTACTCA -3'
	Reverse	5'- TGGTGATCCTCTTGTAGCTCTCC -3'
Tnfa	Forward	5'- GCCACCACGCTCTTCTGCCT -3'
	Reverse	5'- GGCTGATGGTGTGGGTGAGG -3'
Il1b	Forward	5'- TGCAGAGTTCCCCAACTGGTACATC -3'
	Reverse	5'- GTGCTGCCCTAATGTCCCCTTGAATC -3'
Hk2	Forward	5'- GCCAGCCTCTCCTGATTTTAGTGT -3'
	Reverse	5'- GGGAACACAAAAGACCTCTTCTGG -3'
16s	Forward	5'- CCGCAAGGGAAAGATGAAAGAC -3'
	Reverse	5'- TCGTTTGGTTTCGGGGTTTC -3'
Pgc1a	Forward	5'- TATGGAGTGACATAGAGTGTGCT -3'
	Reverse	5'- CCACTTCAATCCACCCAGAAAG -3'
18s	Forward	5'- CTCAACACGGGAAACCTCAC -3'
	Reverse	5'- CGCTCCACCAACTAAGAACG -3'

Table S2. List of primary antibodies used for immunoblot analysis

Target protein	Catalog number	Working dilution	Company
p-S6K1 (Thr389)	2708	1:1000	Cell Signaling Technology (Danvers, MA, USA)
S6K1	9205	1:1000	Cell Signaling Technology (Danvers, MA, USA)
p-4EBP1 (Thr37/46)	2855	1:1000	Cell Signaling Technology (Danvers, MA, USA)
4EBP1	9452	1:1000	Cell Signaling Technology (Danvers, MA, USA)
Puromycin	MABE343	1:1000	Merck (Darmstadt, Germany)
P21	sc-6246	1:1000	Santa Cruz Biotechnology (Dallas, TX, USA)
P16	sc-166760	1:1000	Santa Cruz Biotechnology (Dallas, TX, USA)
OXPHOS complex	ab110413	1:1000	Abcam (Cambridge, UK)
Mfn2	sc-100560	1:1000	Santa Cruz Biotechnology (Dallas, TX, USA)
Drp1	5391	1:1000	Cell Signaling Technology (Danvers, MA, USA)
Fis1	PA1-41082	1:1000	Thermo Fisher Scientific (Waltham, MA, USA)
ZO-1	sc-10804	1:1000	Santa Cruz Biotechnology (Dallas, TX, USA)
Occludin-1	sc-5562	1:1000	Santa Cruz Biotechnology (Dallas, TX, USA)
Cloudin-1	sc-166338	1:1000	Santa Cruz Biotechnology (Dallas, TX, USA)
TNF-α	ab66579	1:1000	Abcam (Cambridge, UK)
COX-2	12282	1:1000	Cell Signaling Technology (Danvers, MA, USA)
β-actin	sc-47778	1:1000	Santa Cruz Biotechnology (Dallas, TX, USA)

Table S3. Pairwise PERMANOVA comparisons of gut microbial beta diversity using weighted and unweighted UniFrac distances among experimental groups

**Weighted**						
**Pairs**	**Df**	**SumsOfSqs**	**F.Model**	**R2**	**p.value**	**p.adjusted**
**R1 vs P8**	1	0.067841	1.678876	0.218636	0.157	0.471
**R8 vs P8+*Akk***	1	0.032088	1.37207	0.186117	0.249	0.747
**R1 vs P8+*Akk***	1	0.084277	2.488418	0.293155	0.099	0.297
**Unweighted**						
**Pairs**	**Df**	**SumsOfSqs**	**F.Model**	**R2**	**p.value**	**p.adjusted**
**R1 vs P8**	1	0.133499	1.765565	0.227358	0.026	0.078
**R8 vs P8+*Akk***	1	0.092811	1.29617	0.177651	0.097	0.291
**R1 vs P8+*Akk***	1	0.129118	1.963264	0.24654	0.045	0.135

Reference

1. Choi PG, Park SH, Jeong HY, Kim HS, Hahm JH, Seo HD*, et al.* 2024. Geniposide attenuates muscle atrophy via the inhibition of FoxO1 in senescence-accelerated mouse prone-8. *Phytomedicine* **123:** 155281.
